# DIODEM – A Diverse Inertial and Optical Dataset of kinEmatic chain Motion

**DOI:** 10.1038/s41597-025-05468-w

**Published:** 2025-07-23

**Authors:** Simon Bachhuber, Dustin Lehmann, Ive Weygers, Thomas Seel

**Affiliations:** 1 Leibniz Universität Hannover, Institute of Mechatronic Systems, Hannover, 30167 Germany; 2 Technische Universität Berlin, Control Systems Group, Berlin, 10587 Germany; 3 FAU Erlangen-Nürnberg, Department Artificial Intelligence in Biomedical Engineering, Erlangen, 91052 Germany

**Keywords:** Biomedical engineering, Scientific data, Mechanical engineering

## Abstract

Inertial Motion Tracking (IMT) faces critical challenges including magnetometer-free sensing, sparse sensor configurations, sensor-to-segment alignment, and motion artifact compensation. Current IMT algorithms require systematic evaluation across combinations of these challenges in controlled environments with accurate ground truth data. This paper presents DIODEM–a comprehensive dataset comprising 46 minutes of synchronized optical and inertial data from five-segment Kinematic Chains (KCs). The dataset features 20 markers and ten IMUs (both rigidly and foam-attached) across two distinct kinematic configurations: an “arm” chain with hinge and spherical joints, and a “gait” chain with hinge and saddle joints. The KCs perform diverse motions including random movements at various speeds, pick-and-place tasks, and gait-like patterns. Key technical contributions include: (1) mechanically controlled setup with known kinematics, (2) systematic inclusion of motion artifacts through foam-attached IMUs, (3) diverse joint types including 1D, 2D, and 3D joints, and (4) comprehensive motion variety supporting sparse sensing scenarios. The dataset enables researchers to systematically study individual and combined IMT challenges, facilitating algorithm development for applications ranging from biomechanics to autonomous systems.

## Background & Summary

Numerous engineering applications require precise tracking of articulated bodies in KCs. Notable examples include: Unobtrusive tracking of human motions outside laboratory environments^1,2^, integrating with robotic systems in immersive industrial or clinical environments^3^ and creating real-time feedback mechanisms in rehabilitation^4^, and aerial robotics^5^. In all of these and many more applications, inertial measurement units (IMUs) play a crucial role for their ability to estimate the complete pose of KCs – providing a cost-effective and reliable alternative to state-of-the-art multi-camera and marker-less^6,7^ alternatives that constantly require line of sight.

Recent developments have significantly enhanced the practical application potential of IMT by addressing several inherent challenges: Firstly, the accuracy of orientation estimation has been substantially improved through the adoption of neural network-based approaches^8^ and advanced complementary filter structures^9^. Secondly, most indoor environments exhibit a disturbed magnetic field^10,11^, which has led to the development of numerous joint-specific magnetometer-free motion tracking solutions^12–14^. Furthermore, application-specific solutions have been proposed to achieve plug-and-play sensor-to-segment alignment^15–17^, and assignment^18–20^.

Although many solutions are available for specific IMT challenges underlying a practical use-case, to truly advance IMT in real-world applications, solutions for combinations of IMT challenges need to be developed. Furthermore, real-world applicability of IMT calls for several additional vastly understudied IMT challenges, such as sparse sensing^21–23^ (where fewer sensors than segments are employed) and effective strategies for mitigating artifacts (caused by the nonrigid attachment of sensors relative to the bone due to soft-tissue motion)^24,25^. Combining IMT challenges is still largely unexplored, and addressing them holistically could significantly impact the practical deployment and effectiveness of IMT.

The growing number of recently published datasets that include inertial sensors underscores the expanding interest in this area. This includes datasets, focused on specific use cases in human motion tracking^26–31^ and datasets that bring novel measurement modalities^32–36^ to the forefront. However, these datasets involve skin-attached sensors and human kinematics which introduce inaccuracies in the ground truth kinematics due to soft-tissue motion, errors in sensor-to-segment alignment, and human joint inaccuracies unless markers are drilled to the bone^36^, and that these effects can be entangled inseparably. Compared with these datasets, the dataset proposed here is designed to catalyze further advancements in IMT by utilizing a mechanical setup which allows for a controlled environment with accurately known kinematics and ground truth, and well-calibrated sensor attachments. To the best of our knowledge, there exists only a single dataset^37^ that also uses a mechanical setup. It uses a motor-driven double-hinge-joint three-segment KC that performs a limited number of swinging motions and excludes all forms of global KC translation and/or rotation. In contrast, the proposed dataset offers long five-segment KCs interconnected by hinge-, saddle-, and spherical joints with rigidly and foam-attached IMUs that performs motions of various speeds and characteristics including global KC translation and/or rotation. Due to the large number of segments, as well as the different types of joints, motions, and sensor attachments, a large variety of fundamental underlying Inertial Motion Tracking Problems (IMTPs) and combinations of IMT challenges can be created such as, e.g., different kinematic structures and lengths, Degrees of Freedom (DoF), and number of sensors; including the ability to modify levels of sparseness and introduce motion artifacts. Additionally, we provide easy-to-use functions to define specific IMTPs tailored to the envisioned use-case. Overall, this results in a large set of valuable real-world IMTPs that can be systematically studied and solved in a controlled environment, thus evaluating the practical applicability of inertial sensors in diverse settings and applications.

## Contributions & Innovations

The DIODEM dataset addresses critical gaps in IMT research by providing the first comprehensive, mechanically controlled dataset that enables systematic study of combined IMT challenges. Our key contributions are:

1. **Mechanically Controlled Ground Truth:** Unlike existing datasets that rely on human subjects with inherent kinematic uncertainties, we developed a precision-engineered mechanical setup using 3D-printed five-segment KCs. This approach provides: 1) accurately known kinematics without soft-tissue motion artifacts that confound human-based datasets, 2) repeatable joint constraints with validated 1D hinge joints, 3) controlled experimental conditions enabling systematic algorithm evaluation.
2. **Comprehensive Joint Type Coverage:** We systematically include diverse joint configurations rarely available in existing datasets including: 1D joints (hinge joints) oriented along x-, y-, and z-axes; 2D joints (saddle joints) enabling two rotational DoF, as well as 3D joints (spherical joints) allowing full rotational freedom. We provide mixed configurations via two distinct KCs combining different joint types.
3. **Systematic Motion Artifact Investigation:** We provide the first dataset to systematically compare rigid and non-rigid IMU attachments. A dual IMU per segment configuration, i.e., every segment is equipped with both a rigidly-attached and a foam-attached IMU, allows to quantify motion artifacts. We provide a first spectral analysis that compares the frequency spectrum of both IMU attachments.
4. **Comprehensive Motion Diversity:** Our dataset includes broad motion variety spanning practical application scenarios:
  Canonical motions: Systematic excitation of individual DoF
  Application-specific motions: Pick-and-place and gait-like movement patterns
  Speed variations: From quasi-static to fast dynamic movements
  Global translations: Both motion scenarios with and without global translation of the KCs
5. **Enabling Framework for Combined Challenge Studies:** We provide tools and data organization that enables researchers to systematically study combinations of IMT challenges: by omitting individual IMU measurements, researchers can study sparse sensing scenarios. Furthermore, researchers can study magnetometer-free operation as complete 3-axis gyroscope and accelerometer data is provided. Researchers can perform sensor misalignment studies by comparing aligned to deliberately misaligned configurations. Lastly, we provide an easy-to-use Python package for simplified data access.

## Methods

In this section, we describe the experimental setup and the recorded data. Specifically, we first outline the measurement setup, then describe the measurement protocol – including the sequence of actions that are performed – and then the variables that are altered in order to create a set of different experiments. Finally, we describe the processing that is applied to all experimental recordings.

### Measurement Setup

**Fig. 1:**
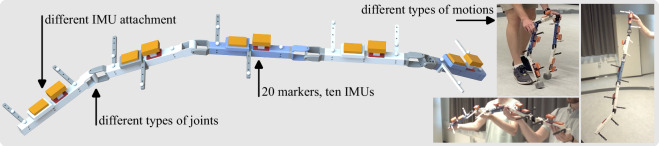
This dataset contains optical and inertial data of five-segment KCs that perform motions of various speeds and characteristics. This figure shows the CAD model of one of the experimental 3D-printed five-segment KC, titled arm, with ten IMUs (orange boxes) and 20 optical motion capture markers (gray spheres). The KC uses three hinge joints, each oriented along the z, y, and x axes, respectively, followed by a spherical joint. In addition, the types of joints can be modified to create a second distinctive five-segment KC, titled gait, and experimental trials have been conducted for both KCs. All segments of both KCs are equipped with two IMUs: one firmly attached to the segment and another affixed nonrigidly using foam padding.

In all experiments, a 3D-printed five-segment KC moves in space (see Fig. 1). We have recorded the motion of two five-segment KCs that differ in the types of joints used. The first KC, titled arm, consists of three hinge joints, each oriented along the z, y, and x axes, respectively, followed by a spherical joint. The second KC, titled gait, consists of a sequence of y-hinge, saddle, saddle, and y-hinge joints. The majority of the parts of the KC are 3D-printed using PLA on a Prusa MK4 printer. The joints use additional off-the-shelf hardware such as bearings for smooth movement. The CAD files are made available^38^. For both KCs, all segments are of equal physical dimensionality and only differ in their coloring and marker-attachment side beams.

**Fig. 2:**
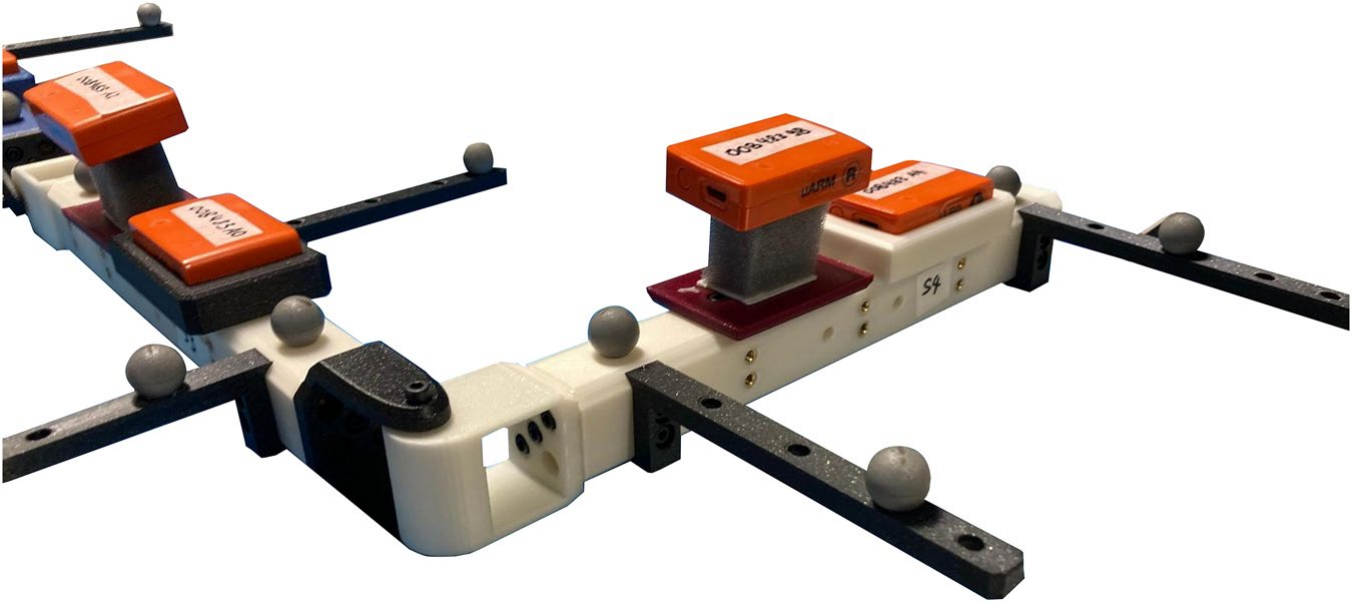
Each segment of both five-segment KCs is equipped with two IMUs: one firmly attached to the segment and another affixed nonrigidly using foam padding. The foam padding allows for relative motion between the segment and the sensor, which creates motion artifacts, and its compensation requires advanced IMT solutions.

To each segment in both five-segment KCs, two IMUs and four Optical Motion Capture (OMC) markers are attached. The two wireless IMUs per segment (Mtw Awinda, Xsens, Enschede, the Netherlands) record 3D acceleration, angular velocity, and magnetic field density at a sampling rate of 40 Hertz. One IMU is attached rigidly to a 3D-printed fixture. The second IMU is attached nonrigidly to the segment via foam, see Fig. 2. During recording, all ten IMUs, comprising two units per segment across five segments, wirelessly transmit their data to a host PC using proprietary software. Note that both IMUs are attached such that the IMU’s sensing x-axis aligns with the axial/longitudinal direction of the segment, and in a way that both IMUs are aligned with the segment’s coordinate system as defined by the OMC markers.

Four OMC markers are rigidly attached either directly to each segment, or on 3D-printed beams that point orthogonal to the segment’s longitudinal direction. For each segment, the four markers are placed specifically to allow for simple and robust calculation of an orthogonal coordinate system. A minimum of three non-colinear markers are necessary to establish a coordinate system, but four markers per segment were used to reduce registration errors from occlusion in the optical motion tracking system. The 3D position of all 20 markers (five segments, four markers per segment) are tracked using an OMC system (OptiTrack Prime 22, NaturalPoint, Inc., Corvallis, U.S.A.) with an array of twelve cameras and at a sampling rate of 30 Hertz.

Additionally, video recordings were obtained using two high-definition cameras (OptiTrack Prime Color) from two different angles for transparent documentation, understanding and referencing of the applied measurement protocol.

### Measurement Protocol

For both five-segment KCs several experiments have been performed. An experiment corresponds to a type (or several types of) motion(s) that are sequentially performed in one continuous recording session. For each experiment, the data was obtained through the following actions: 1) The two synchronized cameras start recording; 2) The ten synchronized IMUs start recording; 3) The OMC starts recording (20 synchronized markers are tracked); 4) The five-segment KC rests for 30 seconds (this enables the users to perform bias estimation and removal if desired, or to validate such methods); 5) The five-segment KC moves in space; 6) The OMC stops recording; 7) The ten IMUs stop recording simultaneously; 8) The two cameras stop recording simultaneously.

### Measurement Variables

In total, eleven experiments are obtained. Each experiment uses the hardware and the measurement protocol as described above. Recall that each experiment consists of a five-segment KC that is manipulated by two human motion tracking experts to create a sequence of motions, e.g., the KC *arm* performs first 30 seconds of slow random motion, then pause for ten seconds, and continue and finish the experiment with 30 seconds of fast random motion. The wording of slow and fast motion is here used to identify the type of motion. The eleven experiments are created by varying the five-segment KC between the arm and gait chain, and by varying the sequence of types of motion between qualitatively distinctive types of motion. The types of joints of the two KCs have been chosen to allow for motion that is inspired by upper-body pick-and-place motion and by lower-body gait-like motion, respectively. The types of motions are classified as motion without pattern (random motion) and with pattern. For both of these types of motion, the excitation level is varied from standstill up to very fast motion. Additionally, distinctive types of motion, such as seesawing or shaking, are recorded. Regarding motions with pattern, distinctive types of motion, such as pick-and-place and gait-like motion, are recorded. Moreover, a large number of types of motions are recorded in two variations, once where the KC is undergoing large amounts of (global) translation, and once where the amount of (global) translation is minimized. Overall, the following types of motions are recorded:

- Canonical: Excite all DoF of the KC consecutively, as decoupled as possible, at approximately one-second intervals. For example, a hinge joint is moved across its entire range of motion spectrum in about one second. Then there is a short pause and the next joint is excited.
- Freeze: The KC remains as still as possible for a few seconds (less than three seconds) and is “frozen” in motion.
- Pause: The KC rests on the table and is at a complete standstill.
- Slow: The KC is slowly moved randomly in space and all DoF are excited as evenly as possible. The KC is not deliberately moved and rotated globally in space.
- Fast: The KC is quickly moved randomly in space and all DoF are excited as evenly as possible. The KC is not deliberately moved and rotated globally in space.
- Dangle: The KC is only held at one segment and hangs downwards. This segment is moved and the rest of the KC follows physically and is in free fall.
- Global: The KC is deliberately moved and rotated globally in space.
- Shaking: The KC is not rotated but rather quickly translated by shaking the KC.
- Pick-and-place: The KC is moved such that it mimics arm motion that performs a pick-and-place task.
- Gait: The KC is moved such that it mimics the motion of the lower-body during gait. Gait-like motion is performed to achieve a faster or a slower gait.
- Quasi-static: The KC is very slowly moved randomly in space and all DoF are excited. The KC is not deliberately moved and rotated globally in space.

### Data Processing

All experiments are processed using the same pipeline that includes the following steps:

1. Replacing NaN values in the marker trajectories obtained from the OMC system using cubic interpolation. This method ensured that the data remained consistent and accurate for further analysis.
2. Time-synchronization of inertial and optical data by cropping of initial inertial data. This is because, as specified in the measurement protocol, the IMUs starts recording before the OMC system starts recording. The cutoff point is found using the method as proposed in^39^ which relies on cross-correlation of calculated (OMC data) and measured (IMU data) angular velocities.
3. Matching data dimensions, by cropping inertial data to match the (now synchronized) optical data.
4. Construction of an orthogonal body coordinate system ${\mathcal{B}}$ using the four marker trajectories for each segment. Figure 3 shows the different local coordinate systems and reference coordinate systems. This is done by first estimating the plane that is spanned by the four OMC markers (to this end, at least three markers are required; must be available and not NaN). Afterwards, the cross product is used to find the normal direction of the plane. Then, from the plane information (x-y-plane) and normal direction (z-direction) an orthogonal coordinate system ${\mathcal{B}}$ that aligns with the respective sensor coordinate system ${\mathcal{S}}$ is constructed.

**Fig. 3:**
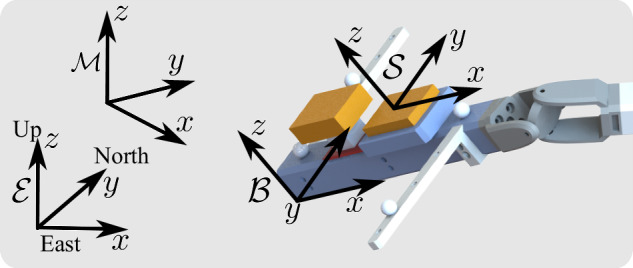
Definitions of the different local coordinate systems and reference coordinate systems. IMT methods use the IMU data (orange boxes) as measured in the local sensor coordinate system ${\mathcal{S}}$ to estimate the orientation of the local sensor coordinate system relative to the earth reference coordinate system ${\mathcal{E}}$ with an x-axis pointing towards east, a y-axis pointing to the magnetic north, and a z-axis pointing upwards. The OMC system tracks the 3D position of the markers (white spheres) relative to the OMC’s reference coordinate system ${\mathcal{M}}$. The markers are rigidly attached to the segment and their 3D position trajectories can be used to span an orthogonal coordinate system for the segment (or body) ${\mathcal{B}}$.
5. Correction of any misalignment errors between the local sensor coordinate system ${\mathcal{S}}$ and the body coordinate system of the respective segment ${\mathcal{B}}$ which has been constructed by the OMC marker trajectories in the previous step. This is done by optimizing for a sensor-to-segment rotation such that the rigidly-attached IMU measurements match a virtual IMU that follows the OMC coordinate system. The utilized procedure for coordinate system alignment is outlined in detail in Appendix C, equations (A5)-(A7), in^39^. The determined sensor-to-segment rotation is then used to transform all IMU measurements from the local sensor coordinate system ${\mathcal{S}}$ to the body coordinate system ${\mathcal{B}}$.
6. Alignment of the earth reference coordinate system ${\mathcal{E}}$ and the OMC reference coordinate system ${\mathcal{M}}$. The OMC system records the marker trajectories in an internal, arbitrary reference coordinate system ${\mathcal{M}}$. Consequently, the constructed body coordinate system ${\mathcal{B}}$ captures the orientation of the segment relative to the OMC’s reference coordinate system ${\mathcal{M}}$. However, the IMUs measures the magnetic field and gravity vector which are defined relative to the earth reference coordinate system ${\mathcal{E}}$. In this last preprocessing step, we compute the constant rotation between OMC’s reference coordinate system ${\mathcal{M}}$ and earth reference coordinate system ${\mathcal{E}}$. This computation is performed simultaneously with the optimization of the previous step. This method is outline in detail in^39^. The determined rotation between the reference coordinate systems is then used to transform all 3D marker trajectories and body coordinate systems from ${\mathcal{M}}$ to ${\mathcal{E}}$.

For more comprehensive details regarding these preprocessing steps and the software implementations utilized, readers are referred to the study by^39^. The data processing software is made openly available^38^.

## Data Records

The data records are hosted in the following repository: 10.7910/DVN/SGJLZA^38^.

A secondary host, in case the primary host is offline, is available at 10.5281/zenodo.15511879. The data records include: 1) the (processed) dataset, 2) associated videos of the experiments and images of the KCs, 3) the corresponding raw data and data processing software and tools employed, and 4) the CAD files for 3D-printing the KCs. The subsequent subsections are structured accordingly.

The folder structure of the data records is outlined in Table 2.

### Processed Dataset /dataset/*

The dataset is contained in the folder /dataset/*. It contains optical and inertial data of a five-segment KC moving in space. There exist two different five-segment KCs, namely arm and gait. For both KCs, each experiment is a sequence of several types of motions motionY, and several experiments expX (in total eleven, E01 to E11) are obtained by varying the types of motion that are performed. The total lengths of the eleven experiments are given in Table 1, and the dataset’s folder structure is outlined in Table 2.

**Table 1:** Summary of processed dataset.

Experiment →	E01	E02	E03	E04	E05	E06	E07	E08	E09	E10	E11	∑
KC	Arm	Arm	Arm	Arm	Arm	Gait	Gait	Gait	Gait	Gait	Gait	2
Duration [seconds]	368	350	275	207	80	294	359	277	212	151	205	2778
Number of Motions	13	8	10	8	2	12	8	9	8	3	7	88

In total, eleven experiments are obtained. Five experiments use the five-segment KC *arm* and six experiments use the five-segment KC *gait*. Each experiment is typically several minutes in duration and consists of various types of motions. For all experiments and all motions therein, the same extensive inertial and optical data is available^38^ which consists of ten 9D (3D accelerometer, 3D gyroscope, 3D magnetometer) IMUs, 20 3D OMC marker trajectories, and five OMC orientation trajectories. Additionally, for documentation purpose, there is video recording at all times from two camera angles.

**Table 2:** Overview of the folder structure of the data records^38^.

	

The data records include 1) the (processed) dataset, 2) associated videos of the experiments and images of the KCs, 3) the corresponding raw data and data processing software and tools employed, and 4) the CAD files for 3D-printing the KCs.

For each experiment expX and motion motionY there are three files:

- expX_motionY_imu_nonrigid.csv: The accelerometer, gyroscope, and magnetometer measurements of five IMUs where each IMU is *nonrigidly* attached to the respective segment. These measurements are expressed in the respective body coordinate system ${\mathcal{B}}$.
- expX_motionY_imu_rigid.csv: The accelerometer, gyroscope, and magnetometer measurements of five IMUs, where each IMU is *rigidly* attached to the respective segment. These measurements are expressed in the respective body coordinate system ${\mathcal{B}}$.
- expX_motionY_omc.csv: For each of the five segments, the trajectories of four 3D marker positions expressed in coordinates of the earth reference coordinate system ${\mathcal{E}}$, and the trajectory of quaternions that encodes the orientation of the segment’s body coordinate system relative to the earth reference coordinate system.

For both expX_motionY_imu_nonrigid.csv and expX_motionY_imu_rigid.csv, the header specifies

- the segment to which the IMU is attached,
- the accelerometer, gyroscope, and magnetometer readings,
- the x-/y-/z-component,

and it starts with seg1_acc_x,seg1_acc_y. For expX_motionY_omc.csv, the header specifies

- the segment to which the marker or estimated orientation belongs to,
- which of the four markers it is,
- the x-/y-/z-component (or u-/x-/y-/z- for quaternion),

and it starts with seg1_marker1_x,seg1_marker1_y.

**Fig. 4:**
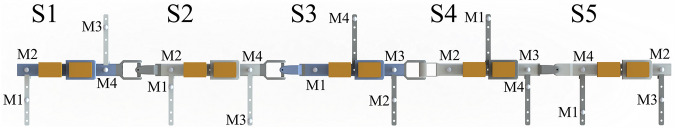
The numbering of segments (S1 to S5) and markers (M1 to M4) here given for the KC *arm* (used in experiments one to five). The types of joints for the KC *arm* (from left to right) are spherical, hinge-x, hinge-y, and hinge-z. The second KC *gait* (used in experiments six to eleven) consists of the segments (from left to right) S5-S1-S2-S3-S4 with the types of joints hinge-y, saddle, saddle, and hinge-y. The marker numbers for each segment are the same for KC *arm* and gait.

For all .csv files, the sampling rate is specified as a comment in the first line, e.g., # sampling frequency: 40. All units are given in seconds/meters/radians or A.U. for the magnetometer. The numbering of segments (S1 to S5) and markers (M1 to M4) for both KCs (arm and gait) are given in Fig. 4. Note that the sequence of types of motions that make up one experiment, they are from one continuous recording session, and as such, they can be concatenated to create a longer time series. Finally, we list the sequences of types of motions of all experiments.

- E01: canonical, pause1, slow1, pause2, fast, pause3, fast_slow_fast, freeze1, fast_slow, freeze2, slow2, shaking, pause4
- E02: canonical1, pause1, slow_fast_mix, pause2, canonical2, pause3, slow_fast_freeze_mix, pause4
- E03: slow1, dangle1, pause1, dangle2, pause2, slow2, dangle3, pause3, dangle4, pause4
- E04: slow_global, pause1, fast_global, pause2, dangle_global1, pause3, dangle_global2, pause4
- E05: pickandplace, pause
- E06: canonical, pause1, slow1, pause2, fast, pause3, fast_slow_fast, freeze1, fast_slow, freeze2, slow2, pause4
- E07: canonical1, pause1, slow_fast_mix, pause2, canonical2, pause3, slow_fast_freeze_mix, pause4
- E08: slow, dangle1, pause1, dangle2, pause2, dangle3, pause3, dangle4, pause
- E09: slow_global, pause1, fast_global, pause2, dangle_global1, pause3, dangle_global2, pause4
- E10: gait_slow, gait_fast, pause
- E11: quasistatic1, slow, rotation, shaking, quasistatic2, explosiv, pause

### Videos /videos/* and Images /images/*

The folder /videos/* contains video recording of all experiments from two camera angles. For all eleven experiments, the video files inside the folder are named expX_camera1.mp4 and expX_camera2.mp4. Additionally, there are rendered videos of all experiments from three different perspectives and they are named expX_render1.mp4, expX_render2.mp4, and expX_render3.mp4. The folder /images/* contains images of the KCs.

### Raw Data and Preprocessing Logic /make_dataset/*

For reproducibility, the folder /make_dataset/raw_data contains the raw data as it is recorded using the hardware of the measurement setup. In combination with published data processing software, this allows any user to exactly reproduce the processed dataset as it is published.

### CAD Files /cad_files/*

Additionally, we publish the files required for the 3D printing of the two five-segment KCs. The CAD files may be used to 3D-print new KCs and extend this dataset to include additional types of joints, motions, or combinations thereof.

### A Broad Range of Inertial Motion Tracking Problems

The richness and versatility of the present dataset is showcased with the great amount of well-controlled IMTPs that can be defined, by selecting specific combinations of the data, and thus varying the following aspects:

- *Types of Joints* (all 1D/2D/3D joints are available): Users of this dataset can create IMTPs that involve 1D, 2D, and/or 3D joints by selecting specific segments. For 1D joints, x-, y-, and z-joint-axes directions are available. As a first step, users identify the desired type of joint in Fig. 4 and select the adjacent segment numbers. For example, for a two-segment KC with a 2D joint, users have to choose the KC *gait*, and then can chose between the segments S2-S3 or S5-S1. For the KC *gait*, users can then choose between E06 to E11 (e.g., see Table 1) and from all motions therein.
- *Length of KC* (can be varied up to a length of five segments): Users can create IMTPs that involve a single segment or KCs of length two, three, four, or five segments. The desired sub-chains can be selected (Fig. 4) and the desired segment numbers can be identified.
- *Sparse Sensing* (every segment has IMU data available that may optionally be dropped): Users can create IMTPs that use only a limited number of IMUs. This can be easily achieved by dropping the respective IMU measurements. For example, for the tracking of a three-segment KC with double hinge joints, users can identify (Fig. 4) that either S1-S2-S3 or S2-S3-S4 are a suitable combination of segments, both from the KC *arm*. Then, users can choose between E01 to E05 and choose from any motion therein. Finally, to achieve sparse sensing the IMU measurements of the middle segment, so either S2 or S3 are dropped.
- *Magnetometer-free Sensing*: Users can create IMTPs that do not make use of the magnetometer reading. This can be achieved by simply dropping the magnetometer measurement.
- *Sensor-to-segment Alignment*: Users can choose to provide the joint-axes direction of 1D joints or not. These joint-axes direction have been validated to be accurate (see Technical Validation).
- *Types of Motions* (diverse set of performed motions): Users can select from a broad range of available motions as listed above.
- *Motion Artifacts*: Users can select between rigidly or nonrigidly attached IMUs for every segment. This choice has been validated to be significant (see Technical Validation).
- *Changing Setup*: Users can create a smooth transition between IMTPs, e.g., the transition between a non-sparse IMTP to a sparse IMTP. This can be relevant for, e.g., addressing dynamic changes in IMU configurations or IMU failures.
- *Trial Duration*: Users can select individual motions or concatenate several motions from one experiment to create a longer time series in a seamless way (without a non-smooth transition). This enables evaluating long-term stability.

### Minor Irregularities in this Data Publication

For completion, we list all the minor irregularities in the provided data with this publication:

- Some of the experimental video recordings contained in the folder /videos/* are slightly out of focus.
- In experiment one (E01) the nonrigid IMU of segment three (S3) is very loosely attached and eventually flips over by 90 degrees along the longitudinal axis of the segment after five minutes and 25 seconds. Whilst inconsistent with the designed measurement protocol, this scenario opens up for evaluating the ability of IMT solutions to detect and compensate attachment loosening.

## Technical Validation

In the following we validate the synchronization between the optical and inertial data, the sensor alignment, the mechanical validity of the joints’ DoF, and investigate the effect of the foam attachment of IMUs by comparing their measurement signals to the signals from the rigidly-attached IMUs.

### Validation of Time Synchronization

The OMC system and IMUs are time-synchronized in the following way: We first pinpoint the IMU data frame corresponding to OMC activation, and 2) trim the trailing portion of IMU data to match the length of the OMC time series. The optimal IMU frame coinciding with OMC activation is determined through cross-correlation between gyroscope and OMC measurements^39^. Table 3 lists for all experiments and all ten IMUs this optimal IMU frame. Since the ten IMUs are software time-synchronized, ideally, the optimal IMU frame should be consistent across IMUs. This is almost perfectly true for all experiments.

**Table 3:** IMU Frame Offset Across Experiments.

Experiment →	E01	E02	E03	E04	E05	E06	E07	E08	E09	E10	E11
IMU 9F	156	112	124	121	107	90	102	96	118	155	70
IMU B8	156	112	124	122	107	92	102	96	118	155	70
IMU A3	156	112	124	121	107	90	102	96	118	155	70
IMU 84	156	112	124	122	108	91	102	96	118	155	70
IMU A0	156	112	124	122	107	91	102	96	118	155	70
IMU A2	156	112	125	121	107	91	102	95	118	156	70
IMU A4	156	112	124	121	107	90	102	96	118	155	70
IMU 9B	156	112	124	122	107	92	102	96	118	156	70
IMU 99	156	112	124	122	107	92	102	96	118	155	70
IMU 85	156	112	124	121	107	91	102	96	118	156	70

### Validation of Alignment of Coordinate Systems

The rigidly-attached IMUs are attached to the segments such that each local sensor coordinate system ${\mathcal{S}}$ is well aligned with the coordinate system ${\mathcal{B}}$ of the segment to which the sensor is attached (see Fig. 3). Because of this hardware alignment, the software alignment that is performed in the data processing (described in the methods section) should only report and correct a small remaining alignment error. The software alignment reports an average alignment correction of 0.56 ± 0.27 degrees (averaged over all ten IMUs and all experiments). The largest reported alignment correction is 1.01 degrees.

### Validation of Mechanical Hinge Joints and of Calibration

There are in total five 1D joints, mechanical hinge joints, in the two five-segment KCs. The KC *arm* has the x-, y-, and z-hinge joints, and the KC *gait* has two y-hinge joints (see Fig. 4). The 3D printed setup, mechanical composition and other factors can introduce an error in the known directions of the axes and/or can lead to a slightly varying, non-constant rotation axes. For each hinge joint, we can propose a joint axis direction and then estimate the mean angle of the residual rotation (MARR), i.e. the rotation that is not around this joint axis direction. We achieve this by computing the relative orientation of the two adjacent segments for the chosen hinge joint from optical data, and then projecting this relative orientation into the rotational feasible subspace as defined by the proposed joint axis direction. The MARR is averaged over all available data for the chosen hinge joint. In addition, for each hinge joint, we can compute the MARR for the respective x-/y-/z-unit vectors, and we can find the optimal joint axis direction that minimizes the MARR. Both values are reported in Table 4. The optimized MARRs are low which validates that the mechanical construction of the 1D joints rotates nearly only around a constant joint axis direction. By comparing the MARR to the optimized MARR and observing that there is almost no difference, we can conclude that the joint axes directions are well calibrated.

**Table 4:** Mean Angle of Residual Rotation (MARR) for all experimental hinge joints and for both major and optimized joint axis direction.

Hinge Joint	Axis Direction	MARR [deg]	Optimized MARR [deg]
KC *Arm*: Between S2 and S3	(1, 0, 0)^⊤^	3.44 ± 1.41	3.42 ± 1.42
KC *Arm*: Between S3 and S4	(0, 1, 0)^⊤^	0.98 ± 0.99	0.98 ± 1.00
KC *Arm*: Between S4 and S5	(0, 0, 1)^⊤^	0.57 ± 1.79	0.55 ± 1.79
KC *Gait*: Between S5 and S1	(0, 1, 0)^⊤^	1.69 ± 3.22	1.63 ± 3.22
KC *Gait*: Between S3 and S4	(0, 1, 0)^⊤^	0.98 ± 0.58	0.95 ± 0.60

Ideally, the MARR should be zero, as it would imply a perfect 1-DoF joint.

### Validation of Rigid and Nonrigid IMU Attachment

**Fig. 5:**
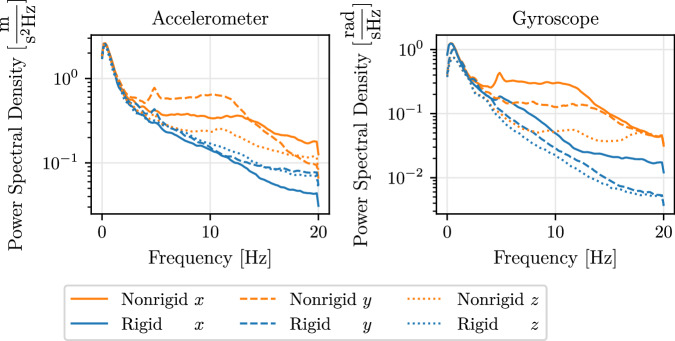
The impact of the foam-attachment of IMUs on the spectral density of the measured signals. Averaged over  ≈ 4 hours of IMU data. We observe that the nonrigid attachment of IMUs does not result in a distinct or narrow range of disturbed frequencies.

In order to assess the impact of the foam attachment of IMUs on the measured signals, we compare their power spectral density to the power spectral density of their rigidly-attached counterparts. The power spectral density is particularly suitable for identifying dominant frequencies and comparing the energy present in different frequency bands and it can be estimated numerically using the Welch’s method^40^. Figure 5 shows the power spectral density over frequency for both accelerometer and gyroscope measurements, grouped by rigidly and foam-attached IMUs and averaged over all experimental data. We observe that the nonrigid attachment of IMUs does not result in a distinct or narrow range of disturbed frequencies. On the contrary, the entire mid-to-high frequency spectrum is affected. Therefore, there exists no special-purpose filtering approach to dampen the affected frequency range which justifies a generic approach based on a low-pass filter.

## Usage Notes

To increase ease-of-use and aid fast adoption, we provide a lightweight Python package, titled diodem, that provides quick access to the data^38^. The Python package is hosted on PyPI and can be easily installed with pip install imt-diodem (requires Python ≥ 3.10). The source code of the Python package is hosted in a GitHub Repository (https://github.com/SimiPixel/diodem). The Python package exports only a single function diodem.load_data, and its usage is self-explanatory and is demonstrated in the following quick-start example.



Additionally, the Python Package allows for easy resampling of the optical and inertial data to a common sampling rate. The following second code example showcases how the data for a three-segment, sparse KC with double hinge joints and y- and z-joint axes directions can be obtained. The task of this IMTP is to estimate the rotational state of the three-segment KC from two, rigidly-attached IMU measurements, attached on the two outer segments.



## Data Availability

The raw inertial and optical data^38^ was processed as outlined in the Methods Section (see Data Processing), and both raw data and the corresponding data processing software is made openly available. The processed dataset as it is published can be reconstructed by following the instructions in /make_dataset/readme.md. The published dataset was processed using the following dependencies: numpy 1.26.4, qmt 0.2.4, pandas 2.2.1, scipy 1.12.0, dm-tree 0.1.8.

## References

[1] Picerno, P. *et al*. Wearable inertial sensors for human movement analysis: a five-year update. *Expert Review of Medical Devices* **18**, 79–94, 10.1080/17434440.2021.1988849 (2021). 34601995 10.1080/17434440.2021.1988849

[2] García-de Villa, S., Casillas-Pérez, D., Jiménez-Martín, A. & García-Domínguez, J. J. Inertial sensors for human motion analysis: A comprehensive review. *IEEE Transactions on Instrumentation and Measurement* **72**, 1–39, 10.1109/TIM.2023.3276528 (2023).37323850

[3] Dafarra, S. *et al*. icub3 avatar system: Enabling remote fully immersive embodiment of humanoid robots. *Science Robotics* **9**, eadh3834, 10.1126/scirobotics.adh3834 (2024). 38266102 10.1126/scirobotics.adh3834

[4] Cinnera, A. M. *et al*. Upper limb assessment with inertial measurement units according to the international classification of functioning in stroke: a systematic review and correlation meta-analysis. *Topics in Stroke Rehabilitation* **31**, 66–85, 10.1080/10749357.2023.2197278 (2024). 37083139 10.1080/10749357.2023.2197278

[5] Zhao, M. *et al*. Design, modeling, and control of an aerial robot dragon: A dual-rotor-embedded multilink robot with the ability of multi-degree-of-freedom aerial transformation. *IEEE Robotics and Automation Letters* **3**, 1176–1183, 10.1109/LRA.2018.2793344 (2018).

[6] Kanko, R. M., Laende, E. K., Davis, E. M., Selbie, W. S. & Deluzio, K. J. Concurrent assessment of gait kinematics using marker-based and markerless motion capture. *Journal of Biomechanics* **127**, 110665, 10.1016/j.jbiomech.2021.110665 (2021). 34380101 10.1016/j.jbiomech.2021.110665

[7] Uhlrich, S. D. *et al*. OpenCap: Human movement dynamics from smartphone videos. *PLOS Computational Biology* **19**, 1–26, 10.1371/journal.pcbi.1011462 (2023). 10.1371/journal.pcbi.1011462 PMC10586693 37856442

[8] Weber, D., Gühmann, C. & Seel, T. RIANN—A Robust Neural Network Outperforms Attitude Estimation Filters. *AI* **2**, 444–463, 10.3390/ai2030028 (2021).

[9] Laidig, D., Weygers, I., Bachhuber, S. & Seel, T. VQF: A Milestone in Accuracy and Versatility of 6D and 9D Inertial Orientation Estimation. In *2022 25th International Conference on Information Fusion (FUSION)*, 1–6, 10.23919/FUSION49751.2022.9841356 (2022).

[10] Subbu, K. P., Gozick, B. & Dantu, R. LocateMe: Magnetic-fields-based indoor localization using smartphones. *ACM Trans. Intell. Syst. Technol.* **4**, 73:1–73:27, 10.1145/2508037.2508054 (2013).

[11] de Vries, W. H. K., Veeger, H. E. J., Baten, C. T. M. & van der Helm, F. C. T. Magnetic distortion in motion labs, implications for validating inertial magnetic sensors. *Gait & Posture* **29**, 535–541, 10.1016/j.gaitpost.2008.12.004 (2009). 19150239 10.1016/j.gaitpost.2008.12.004

[12] Laidig, D., Weygers, I. & Seel, T. Self-Calibrating Magnetometer-Free Inertial Motion Tracking of 2-DoF Joints. *Sensors* **22**, 9850, 10.3390/s22249850 (2022). 36560219 10.3390/s22249850 PMC9785932

[13] Köhler, J., Invernizzi, M., Haan, P. D. & Noe, F. Rigid Body Flows for Sampling Molecular Crystal Structures. In *Proceedings of the 40th International Conference on Machine Learning*, 17301–17326 (PMLR, 2023).

[14] Lehmann, D., Laidig, D., Bachhuber, S., Seel, T. & Weygers, I. Magnetometer-free inertial motion tracking of kinematic chains, 10.2139/ssrn.4785077 (2024).

[15] Seel, T., Raisch, J. & Schauer, T. Imu-based joint angle measurement for gait analysis. *Sensors* **14**, 6891–6909, 10.3390/s140406891 (2014). 24743160 10.3390/s140406891 PMC4029684

[16] McGrath, T., Fineman, R. & Stirling, L. An Auto-Calibrating Knee Flexion-Extension Axis Estimator Using Principal Component Analysis with Inertial Sensors. *Sensors* **18**, 1882, 10.3390/s18061882 (2018). 29890667 10.3390/s18061882 PMC6021833

[17] Olsson, F., Kok, M., Seel, T. & Halvorsen, K. Robust Plug-and-Play Joint Axis Estimation Using Inertial Sensors. *Sensors* **20**, 3534, 10.3390/s20123534 (2020). 32580394 10.3390/s20123534 PMC7348773

[18] Weenk, D., Van Beijnum, B.-J. F., Baten, C. T., Hermens, H. J. & Veltink, P. H. Automatic identification of inertial sensor placement on human body segments during walking. *Journal of neuroengineering and rehabilitation* **10**, 1–9, 10.1186/1743-0003-10-31 (2013). 23517757 10.1186/1743-0003-10-31 PMC3651313

[19] Nazarahari, M. & Rouhani, H. Semi-automatic sensor-to-body calibration of inertial sensors on lower limb using gait recording. *IEEE Sensors Journal* **19**, 12465–12474, 10.1109/JSEN.2019.2939981 (2019).

[20] Taetz, B., Lorenz, M., Miezal, M., Stricker, D. & Bleser-Taetz, G. Jointtracker: Real-time inertial kinematic chain tracking with joint position estimation [version 1; peer review: 1 approved with reservations]. *Open Research Europe* **4**, 10.12688/openreseurope.16939.1 (2024). 10.12688/openreseurope.16939.2 PMC11216284 38953016

[21] Bachhuber, S. *et al*. Plug-and-Play Sparse Inertial Motion Tracking With Sim-to-Real Transfer. *IEEE Sensors Letters* **7**, 1–4, 10.1109/LSENS.2023.3307122 (2023).37529707

[22] Eckhoff, K., Kok, M., Lucia, S. & Seel, T. Sparse magnetometer-free inertial motion tracking - a condition for observability in double hinge joint systems. *IFAC-PapersOnLine* **53**, 16023–16030, 10.1016/j.ifacol.2020.12.403 (2020).

[23] Dorschky, E. *et al* Comparing sparse inertial sensor setups for sagittal-plane walking and running reconstructions. *bioRxiv* 2023–05, 10.1101/2023.05.25.542228 (2023). 10.3389/fbioe.2025.1507162 PMC11879983 40046809

[24] Forner-Cordero, A. *et al*. Study of the motion artefacts of skin-mounted inertial sensors under different attachment conditions. *Physiological Measurement* **29**, N21, 10.1088/0967-3334/29/4/N01 (2008). 18401071 10.1088/0967-3334/29/4/N01

[25] Remmerswaal, E., Weygers, I., Smit, G. & Kok, M. Fast relative sensor orientation estimation in the presence of real-world disturbances. In *2021 European Control Conference (ECC)*, 411–416, 10.23919/ECC54610.2021.9654849 (2021).

[26] Palermo, M., Cerqueira, S. M., André, J., Pereira, A. & Santos, C. P. From raw measurements to human pose-a dataset with low-cost and high-end inertial-magnetic sensor data. *Scientific Data* **9**, 591, 10.1038/s41597-022-01690-y (2022). 36180479 10.1038/s41597-022-01690-y PMC9525570

[27] Jayasinghe, U., Hwang, F. & Harwin, W. S. Inertial measurement data from loose clothing worn on the lower body during everyday activities. *Scientific Data* **10**, 709, 10.1038/s41597-023-02567-4 (2023). 37848448 10.1038/s41597-023-02567-4 PMC10582085

[28] Konak, O. *et al*. Sonar, a nursing activity dataset with inertial sensors. *Scientific data* **10**, 727, 10.1038/s41597-023-02620-2 (2023). 37863902 10.1038/s41597-023-02620-2 PMC10589213

[29] García-de Villa, S. *et al*. A database with frailty, functional and inertial gait metrics for the research of fall causes in older adults. *Scientific Data* **10**, 566, 10.1038/s41597-023-02428-0 (2023). 37626053 10.1038/s41597-023-02428-0 PMC10457385

[30] Seong, M. *et al*. Multisensebadminton: Wearable sensor–based biomechanical dataset for evaluation of badminton performance. *Scientific Data* **11**, 343, 10.1038/s41597-024-03144-z (2024). 38580698 10.1038/s41597-024-03144-z PMC10997636

[31] Dong, A. *et al*. A new kinematic dataset of lower limbs action for balance testing. *Scientific data* **10**, 209, 10.1038/s41597-023-02105-2 (2023). 37059747 10.1038/s41597-023-02105-2 PMC10104813

[32] Santos, G., Wanderley, M., Tavares, T. & Rocha, A. A multi-sensor human gait dataset captured through an optical system and inertial measurement units. *Scientific Data* **9**, 545, 10.1038/s41597-022-01638-2 (2022). 36071060 10.1038/s41597-022-01638-2 PMC9452504

[33] Mehdizadeh, S. *et al*. The toronto older adults gait archive: video and 3d inertial motion capture data of older adults’ walking. *Scientific data* **9**, 398, 10.1038/s41597-022-01495-z (2022). 35817777 10.1038/s41597-022-01495-z PMC9272879

[34] Cognolato, M. *et al*. Gaze, visual, myoelectric, and inertial data of grasps for intelligent prosthetics. *Scientific data* **7**, 43, 10.1038/s41597-020-0380-3 (2020). 32041965 10.1038/s41597-020-0380-3 PMC7010656

[35] Grouvel, G., Carcreff, L., Moissenet, F. & Armand, S. A dataset of asymptomatic human gait and movements obtained from markers, imus, insoles and force plates. *Scientific Data* **10**, 180, 10.1038/s41597-023-02077-3 (2023). 36997555 10.1038/s41597-023-02077-3 PMC10063557

[36] Weygers, I. *et al*. Reference in-vitro dataset for inertial-sensor-to-bone alignment applied to the tibiofemoral joint. *Scientific Data* **8**, 208 (2021). 34354084 10.1038/s41597-021-00995-8 PMC8342472

[37] Szczęsna, A. *et al* Reference data set for accuracy evaluation of orientation estimation algorithms for inertial motion capture systems. vol. 9972, 509–520, 10.1007/978-3-319-46418-3_45 (2016).

[38] Bachhuber, S., Lehmann, D., Weygers, I. & Seel, T. DIODEM – A Diverse Inertial and Optical Dataset of kinEmatic chain Motion, 10.7910/DVN/SGJLZA (2024). 10.1038/s41597-025-05468-w PMC12287372 40702014

[39] Laidig, D., Caruso, M., Cereatti, A. & Seel, T. BROAD—A Benchmark for Robust Inertial Orientation Estimation. *Data* **6**, 72, 10.3390/data6070072 (2021).

[40] Welch, P. The use of fast fourier transform for the estimation of power spectra: A method based on time averaging over short, modified periodograms. *IEEE Transactions on Audio and Electroacoustics* **15**, 70–73, 10.1109/TAU.1967.1161901 (1967).

